# Volcanic activity and hazard in the East African Rift Zone

**DOI:** 10.1038/s41467-021-27166-y

**Published:** 2021-11-25

**Authors:** Juliet Biggs, Atalay Ayele, Tobias P. Fischer, Karen Fontijn, William Hutchison, Emmanuel Kazimoto, Kathy Whaler, Tim J. Wright

**Affiliations:** 1 COMET, School of Earth Sciences, University of Bristol, Bristol, UK; 2 Institue of Geophysics, Space Science and Astronomy, Addis Ababa University, Addis Ababa, Ethiopia; 3 Department of Earth and Planetary Sciences, University of New Mexico, Albuquerque, NM USA; 4 Department of Geosciences, Environment and Society, Université libre de Bruxelles, Av. F Roosevelt 50, 1050 Brussels, Belgium; 5 School of Earth & Environmental Sciences, University of St Andrews, St Andrews, UK; 6 Department of Geosciences, University of Dar es Salaam, P. O. Box, 35052 Dar es Saalam, Tanzania; 7 School of GeoSciences, University of Edinburgh, Edinburgh, UK; 8 COMET, School of Earth and Environment, University of Leeds, Leeds, UK

**Keywords:** Tectonics, Volcanology, Natural hazards

## Abstract

Over the past two decades, multidisciplinary studies have unearthed a rich history of volcanic activity and unrest in the densely-populated East African Rift System, providing new insights into the influence of rift dynamics on magmatism, the characteristics of the volcanic plumbing systems and the foundation for hazard assessments. The raised awareness of volcanic hazards is driving a shift from crisis response to reducing disaster risks, but a lack of institutional and human capacity in sub-Saharan Africa means baseline data are sparse and mitigating geohazards remains challenging.

## Abstract (web summary)

New observations of volcanic and magmatic activity in Africa are changing our views of continental rifting and raising awareness of the associated hazards. However, despite a shift from crisis response to reducing disaster risks, limited capacity means mitigating geohazards remains challenging.

## Introduction

**Fig. 1:**
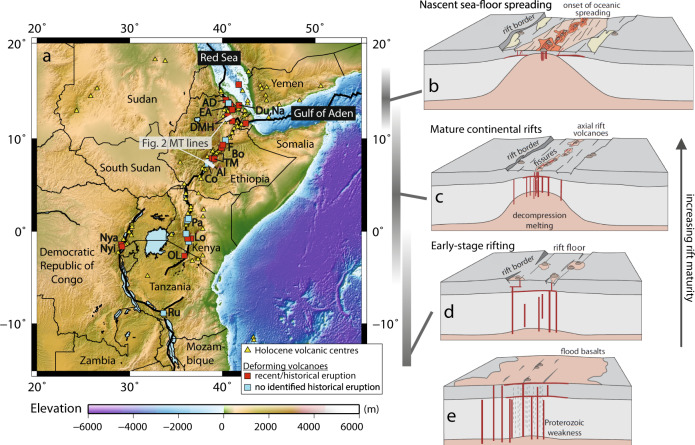
Volcanoes of the East African Rift System. **a** Distribution of volcanoes in the East African Rift, Afar Triangle and Arabian Peninsula from^86^. Symbol shape denotes whether deformation has been reported (square) or not (triangle) from^6^. Colours denotes whether historical eruptions have been reported according to^80^. EA Erte ‘Ale, AD Alu-Dalafilla, Du Dubbi, Na Nabro, DMH Dabbahu Manda Hararo, F Fentale, Bo Boset, TM Tullu Moye, Al Aluto, Co Corbetti, Pa Paka, Lo Longonot, OL Oldoinyo Lengai, Nya Nyamuragira, Nyi Nyiragongo, Ru Rungwe. Updated from ref. ^122^. **b–e** Schematic model of continental rift evolution in the East African Rift System, modified after^8,123^. **b** Nascent sea-floor spreading. **c** Mature continental rifts, with axial rift volcanism forming large silicic centres and basaltic fissures. Extension is accommodated by a combination of faulting and magmatism. **d** Early-stage rifting, with activation of rift border faults, subsidence of a broad zone and diffuse volcanism. **e** Onset of rift formation concurrent with flood-basalt events.

Volcanoes display a diverse range of eruptive styles of varying magnitudes, intensities, and frequencies. A key control on eruptive processes is tectonic setting, which determines how magma is generated, the pathways by which it reaches the Earth’s surface and the characteristics of eruptions. In extensional settings, magma is critical to weakening the lithosphere and allowing continental break-up, and the East African Rift System (EARS) provides a case study for the processes and timescales involved (Fig. 1b–e). Although most regional studies of volcanism have been performed on subduction zones, ~10% of the world’s volcanoes lie in continental rifts including 78 in the EARS (Fig. 1a)^1^. Despite on-going scientific debate regarding the influence of rift dynamics on magmatism e.g. refs. ^2,3^ and over 120 million people living within 100 km of an EARS volcano^1^, rift volcanoes are understudied in comparison to their subduction zone counterparts. Advances in remote sensing are altering our perspective on many aspects of our planet and satellite studies demonstrated that 17 EARS volcanoes experienced periods of deformation during 2003–2008 alone, most of which were previously believed to be quiescent^e.g^^4–6^. These observations raised important questions regarding the processes occurring at rift volcanoes and the associated hazards.

**Fig. 2:**
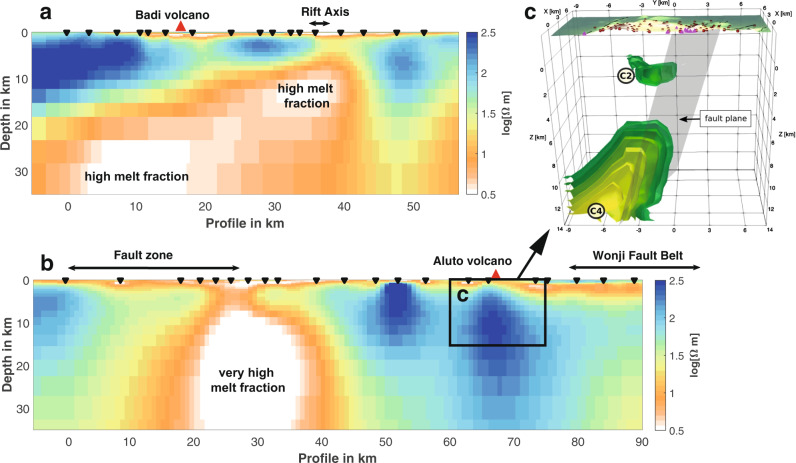
Transcrustal magma storage in a continental rift. **a, b** 2D models of resistivity (inverse of electrical conductivity) along profiles perpendicular to the rift axes. **a** The northern Dabbahu Manda Hararo magmatic segment in Afar^66^ and **b** the central MER (see Fig. 1). Inverted triangles mark the locations of the MT sites projected onto the profiles. Extensive areas of warm to white colours, corresponding to high conductivity, indicate pervasive melt that extends into the upper mantle, and show the offset between lower crustal magma reservoirs and the rift axis in both Afar and MER (Aluto volcano). Moho is at ~22 and 30 km depth beneath Afar and the central MER, respectively^64^. Petrological data suggest the bulk resistivities correspond to at least 12% melt in Afar^66^ and almost 60% beneath the MER^18^. Taken together with other information, we infer a substantial lower crustal off-axis magma reservoir in Afar and considerably higher melt fraction magma focussed into narrow zones of weakness adjacent to the rift border in the MER (Fig. 3). **b** Local-scale 3D MT study of the magmatic system under Aluto volcano showing enhanced electrical conductivity (green-yellow colours) interpreted as a lower crustal zone of melt accumulation (C4) linked to a shallow melt zone (C2) along a fault zone. From ref. ^39^.

The emergent need to develop a holistic understanding of volcanism in the EARS has motivated several multi-disciplinary studies of high-risk volcanoes, approaching the problem from different perspectives. Geophysical techniques including seismology, geodesy, magnetellurics and gravity can be used to image magma storage in the rift and study the short-term processes that drive present activity (Fig. 2). It has been possible to study volcanoes throughout their eruptive cycles, including examples of explosive and effusive eruptions, dyke intrusions, unrest and quiescence. The ability to image the spatial and temporal variations in stress and strain associated with magmatic, hydrothermal and fault systems has provided new insights into the processes of magma storage and ascent. Although the short instrumental record is unable to capture the full range of possible behaviours, the geological record can provide information on the frequency, magnitude and characteristics of past eruptions, and geochemical analyses detail magma storage conditions within the rift.

Finally, high population exposure and vulnerability in Sub-Saharan Africa drive the socio-economic need to understand and manage the potential threats posed by the EARS volcanoes. However, probabilistic volcanic hazard assessment approaches that may be used to inform long-term risk management remain in their infancy in the EARS. Scientific challenges include the sparsity of data and differences in tectonic setting and magma composition, while the lack of historical disasters mean that political awareness is low.

## Magma storage and transport in the East African Rift System

Tectonic activity in East Africa is often attributed to the presence of mantle upwellings at various scales, but the number and depth extent of the upwellings remains debated^7^. The EARS comprises several discrete and diachronous rift sectors, associated with distinct styles of magmatic and volcanic activity (Fig. 1b–e)^8,9^. In this article, we move through the different rift stages by going from the South (early-stage rifting) to the North (sea-spreading) of the EARS. In the early stages of magmatic rifting, volcanism is characterized by unusual magmas and extreme volatile emissions (Fig. 1d). For example, Nyiragongo and its neighbour Nyamuragira are currently the fourth largest non-eruptive emitters of SO_2_ (~ 3500 t/day^10^) globally, and also emit significant quantities of CO_2_ (1 Mt/yr^11^), while Oldoinyo Lengai is the only currently active natrocarbonatite volcano in the world^12^ (Fig. 1a).

The Kenyan Rift and the Main Ethiopian Rift (MER) are mature continental rifts (Fig. 1c), characterized by focussed upwelling and pervasive melt in the upper mantle and lower crust^13,14^. These rifts are dominated by large (5–15 km diameter) silicic caldera systems characterized by post-caldera rhyolitic obsidian domes, flows and pumice cones^15^, interspersed with distributed fields of mafic cones and lava flows^2,16^. Post-caldera peralkaline rhyolites formed by fractional crystallization (>90%) at depths < 6 km (200 MPa) from parental magmas with < 1 wt% H_2_O^17–20^, and large volumes (>100 km^3^) of crystal cumulates are stored as long-lived partially crystalline mush zones with small ephemeral lenses of rhyolitic melt and exsolved volatiles^18,20–22^.

In the Afar region of Northern Ethiopia and Eritrea, the separation of Arabia from Nubia and Somalia over the past 30 Ma has created a region characterized by extensive faulting, lithospheric stretching and, more recently, by intrusive and extrusive magmatic activity, thought to represent the final stages of continental break-up or the onset of sea-floor spreading (Fig. 1b)^23^. Observations of deformation, degassing and seismicity have allowed us to build a picture of the shallow magma storage and transport systems characteristic of the different segments of the EARS.

### Early-stage rifting

**Fig. 3:**
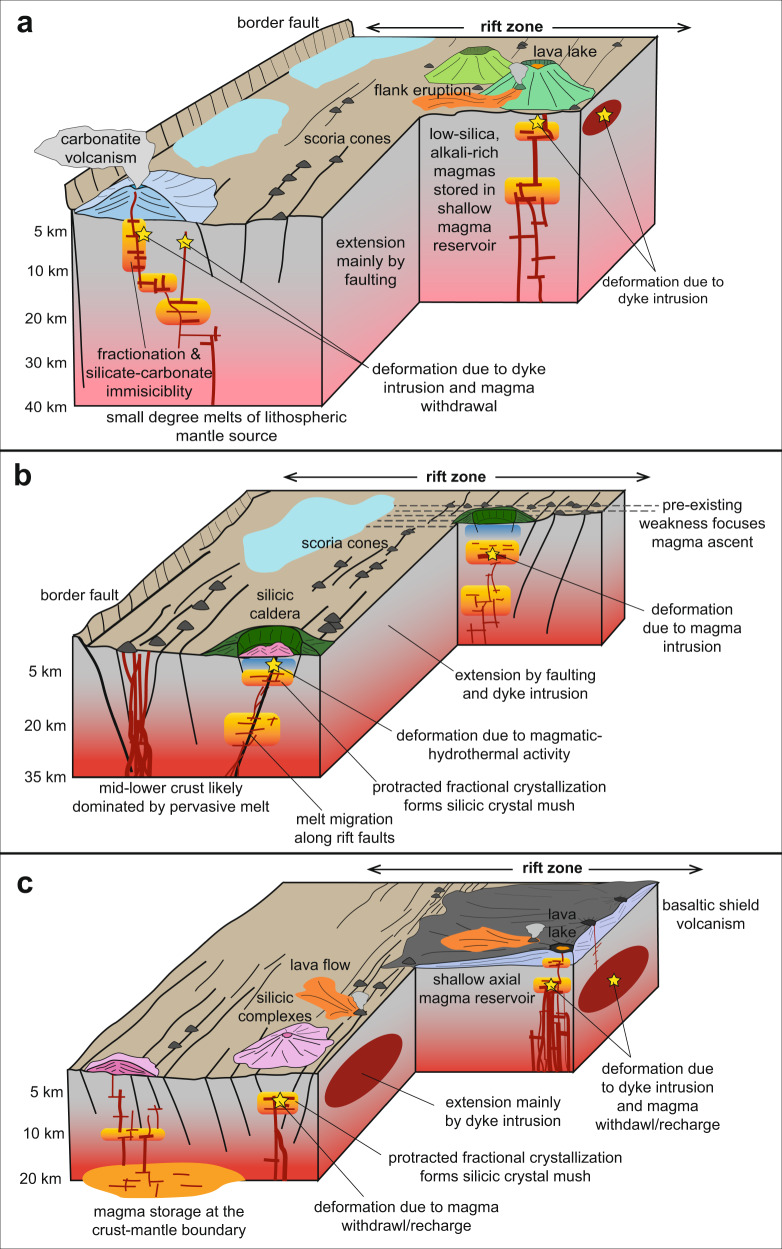
Conceptual model of magmatic systems in rifts at different stages of their evolution. **a** Early-stage rifting, based on Ol Doinyo Lengai and the Natron Rift in Tanzania (foreground), and the Virunga volcanoes on the western branch of the EARS (background). **b** Mature continental rifting, based on the Main Ethiopian Rift. **c** Nascent sea-floor spreading, based on Afar, Ethiopia, with the Manda Hararo Rift in the foreground, and the Erta Ale segment in the background. See text for details.

In the Virunga Volcanic Province in the Democratic Republic of Congo (Figs. 1a, 3a), Nyiragongo hosts one of the world’s longest-lived lava lakes fed by a shallow magma reservoir at ~4 km depth^24^. In 2002, a system of eruptive fractures opened on the southern flank, extending 20 km into the city of Goma, with fast-moving lava flows inundating the city, resulting in ~150 fatalities and leaving ~400,000 people displaced^25^. Deformation patterns indicate that a 40 km-long dyke located at 6 km depth triggered magma injection into a shallow eruptive reservoir, starting 10 months prior to the eruption^26^. More recently, an eruption in May 2021 produced lava flows that reached the city of Goma, prompting another large-scale evacuation. This eruption has also been attributed to a N-S trending dyke^27^. Eruptions of neighbouring Nyamuragira on the other hand, tend to be relatively small and the 2011–2012 event produced only ~0.3 km^3^ of magma^28^. Deformation and petrological studies suggest a source at >10 km depth^29,30^.

At Oldoinyo Lengai in Tanzania (Figs. 1a, 3a), multi-decadal episodes of natrocarbonatite activity, which fill up the summit crater with extremely low-viscous and low-temperature lavas, alternate with explosive eruptions dominated by silicic nephelinite magmas^31^. The natrocarbonatite eruptive phases are volumetrically small compared to the silicic magmatic products^12^, which are thought to contribute the majority of the volatiles emitted by the volcano^32,33^. In 2007–2008, intrusion of a 7–10 km-long dyke less than 20 km from the volcano preceded a new phase of explosive eruptions^34,35^, which generated eruption columns several kms high and spread volcanic ash over the surrounding plains^36^. Recent and extensive seismic studies show that the region is underlain by a complex magmatic plumbing system that extends to about 20 km depth, composed of interconnected layered sills that facilitate the transport of fluids (Fig. 3a)^37^.

### Mature continental rifts

Recent geophysical experiments have enhanced our understanding of the current state of magma storage in mature continental rifts. Magnetotellurics (MT) is widely used in the geothermal exploration industry to identify high electrical conductivity clay caps associated with geothermal alteration^38^ and recent improvements in data coverage and processing methods have enabled both MT and seismic tomography to successfully image the transcrustal magmatic system beneath rift calderas (Fig. 2). At Aluto (Figs. 1a, 3b), this consists of a low-velocity, high conductivity zone of partial (10–15%) melt at depths of 3.5–5 km below the surface^39,40^ and a shallow hydrothermal system (< 3 km) with high shear wave anisotropy and low v_p_/v_s_ consistent with the presence of multiple fracture sets and exsolved gases^40,41^. At Tullu Moye (Fig. 1a), higher conductivity in the zone of partial melt is thought to correspond to higher temperatures and melt fractions (< 33%)^39^. In the lower crust, high electrical conductivities and low seismic velocities indicate a broad zone of partial melt, extending from beneath the rift to the western boundary and in pockets beneath the western plateau, controlled by the topography of the base of the crust^42,43^. Extreme values of seismic anisotropy and v_p_/v_s_ along the NW rift boundary indicate melt is focussed there^44,45^.

Many of the caldera systems in the Main Ethiopian and Kenyan Rifts are experiencing unrest, compatible with the recharge and reorganization of shallow, long-lived mush systems. Deformation has been reported at ten EARS calderas^6^ and temporary seismic deployments have recorded thousands of small magnitude (M < 3) volcano-tectonic earthquakes^46–50^, as well as low-frequency events and swarms characteristic of fluid movement^49^. The spatio-temporal patterns of deformation are highly variable, with footprints ranging from < 1 km^2^ to >150 km^2^ ^6^, but are consistent with sources at the depth of fractional crystallization^4,5,51^. At Corbetti (Figs. 1a, 3b), sustained uplift (>6 cm/yr) has lasted for over a decade (so far) and dynamic gravity measurements indicate a high magma flux accommodated by ductile behaviour^6,51,52^. In contrast, Aluto and Longonot (Fig. 1a) both experienced pulses of rapid uplift (10–15 cm) followed by gradual subsidence, attributed to the interaction between a deep magmatic source and shallow hydrothermal system^5,53^ and at Paka (Fig. 1a), four distinct, but interacting, shallow sources (< 5 km) are required to explain the complex pattern of deformation^54^.

Multiple lines of evidence now agree that the large caldera systems that characterize mature continental rifting are underlain by shallow, restless magma bodies (Fig. 3b). The magmatic systems provide a heat source for abundant hydrothermal systems, giving the EARS an estimated geothermal power potential of >15GWe^55^. Differences in geophysical and petrological observations between systems suggest variations in temperature, composition and structure driven by the complex interplay between the rates of tectonic and magmatic processes^56^.

### Nascent sea-floor spreading

One of the best-studied periods of activity in the Afar region was in 2005–2010 at the Dabbahu Manda Hararo Segment (Figs. 1a, 3c), which was the first subaerial rifting episode where ground movements were mapped with satellite geodesy^57^. A full suite of volcanic rock types ranging from mildly alkaline basalts through to trachyandesite to peralkaline rhyolites have been erupted from the segment, with more silicic products focused around topographically-elevated volcanic centres, separated by regions of intensely fissured and faulted basaltic lava flows^58^. In September 2005, a dyke up to 10 m thick and around 65 km-long intruded in the upper 10 km of crust^59^. The dyke was associated with a small rhyolitic ash eruption at its northern end, but evidence from regional seismicity suggested that the majority of the dyke volume was intruded from a primary source of basaltic composition at the segment centre^60^. Over the next 5 years, a sequence of 13 smaller dykes was intruded along the rift from the segment centre^61^, with local seismic stations able to show that these intrusions had a sudden onset and each took just a few hours to emplace^62^. Three of these smaller dykes breached the surface, erupting basaltic lavas with a transitional sub-alkaline to alkaline composition^63^. Later studies of ground deformation, seismology^64,65^, petrology^3,58^ and MT^66,67^ show that upper crustal magma reservoirs exist beneath the rift axis and silicic centres at 3–5 km depth (Fig. 3c). In contrast, the off-rift Badi volcano (Fig. 2) is underlain by a much larger zone of partial melt that extends into the mantle and is thought to persist for several tens of thousands of years^66^, while the inactive southern part of the segment has small volumes of partial melt in the lower crust (around 20 km depth) adjacent to the rift axis^64,67^.

Further north in Afar, at Erta ‘Ale (Figs. 1a, 3c), the extension rate is slower but the crust is thinner^23,68^ and the rift segment is over-supplied with magma, creating a topographic ridge. At least 4 separate episodes of ground deformation have been observed along the ~115 km-long rift segment since the early 1990s^68–71^. Each of these has involved intrusion along less than 10 km of the rift, with dyke opening confined to the upper few kilometres of crust. The 2008 activity at Alu-Dalafilla (Fig. 1a) was particularly well captured, with two ~2 km-long shallow elongated sill-like magma bodies feeding a dyke and eruption^68^. The most recent eruptive activity on the Erta ‘Ale segment, which began in January 2017, was again consistent with a very shallow connected plumbing system of transitional basaltic composition^72^, with deformation due to a dyke intrusion and eruption being closely tracked by changes in the level of the lava lake^69^, which has been active since at least the 1960s.

Observations of activity in Afar show that dyking rather than faulting is the primary mode for accommodating extension in the elastic upper crust in mature rift systems where sufficient magma supply is available^57^. The behaviour of these magmatic systems is analogous to observations from spreading centres at mid-ocean ridges, where making direct observations of magma dynamics is more challenging. Activity within the Dabbahu Manda Hararo segment is comparable to slow-spreading ridges whereas at Erta ‘Ale the behaviour appears more similar to fast spreading ridges despite the slow rate of extension^57^. Pagli et al.^68^ suggest the surprising behaviour at Erta ‘Ale may be due to the lack of a vigorous hydrothermal circulation, excessive magma production leading to frequent magma chamber replenishment, or could be related to the proximity to the Afar plume.

### Fluid flow

Noble gas isotopes, gas abundance ratios and stable isotopes of C and N unequivocally demonstrate the significance of mantle-derived components to volatile discharges throughout the rift implying that the EARS provides a major pathway for deep earth gases to the atmosphere^73–75^. While these fluids contain C, S, H species and noble gases, most work to date has focussed on quantifying the flux of CO_2_ from the rift system. This requires ground-based spot measurements over strategically selected areas of the vast rift system followed by extrapolation to areas that have not yet been measured. The estimates of CO_2_ flux from the entire eastern branch of the EARS range from 4 to 33 Mt CO_2_/yr^76^ to 71 ± 33 Mt CO_2_/ yr^77^. These large uncertainties demonstrate the challenges to accurately quantifying emissions from large-scale continental rifts and are likely due to the different tectonic settings where the initial measurements have been made. The higher extrapolations^77^ are based on measurements in the Magadi-Natron basin, where remobilized carbon stored under the Tanzanian Craton may contribute to the emissions^78^. Nonetheless, the contribution to the global annual CO_2_ budget from volcanic and active tectonic sources in the region is significant^79^.

As continental rifts develop, the locus of strain accommodation shifts^8^. Measurements of CO_2_ emissions and analyses of gases discharged in springs indicate that in the 3 Ma Natron basin (Northern Tanzania) fluid release is concentrated along the border faults where magma bodies ascend; while in the 7 Ma Magadi basin (Southern Kenya) magmatic volatile release is concentrated along intrarift faults in the centre of the basin. This focussing of magma and fluids towards the rift centre occurs as the rift evolves over time and provides a mechanism that may help weaken the crust and result in the transition to strain accommodation that is more focussed^77^.

**Fig. 4:**
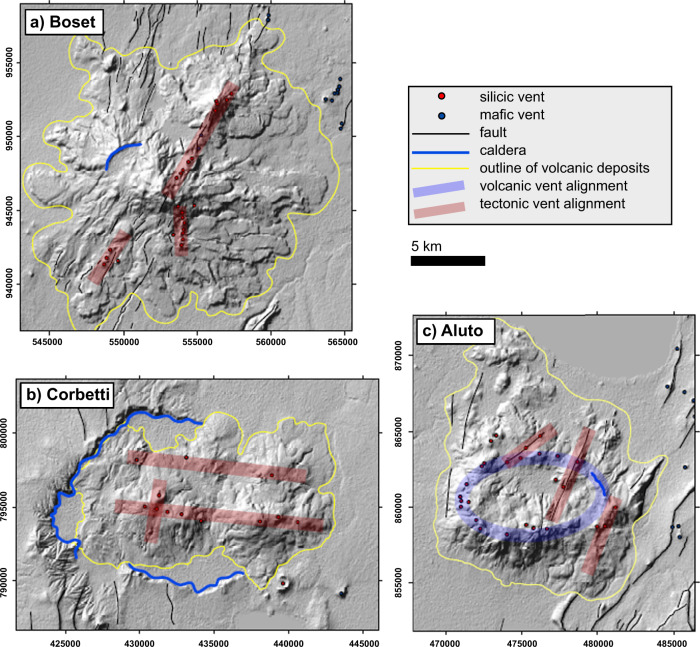
Contrasting examples of the structural controls on post-caldera volcanism. Structural controls are identified by vents, domes and craters in the Main Ethiopian Rift (adapted from ref. ^111^). Volcano locations shown on Fig. 1a. **a** at Boset, trends in vent locations are rift-parallel, aligned with regional faults surrounding the edifice; **b** at Corbetti, post-caldera volcanism is aligned with a pre-existing cross rift structure^84^, **c** at Aluto, vents and craters follow a proposed caldera rim fault^82^, which has now been buried by subsequent eruptions.

On a local scale, the spatial patterns of volcanic activity in the EARS are influenced not only by the regional stress field, but also local stresses and structural controls. Only about a third of the historical eruptive fissures or dykes are parallel to the rift axis, and large edifices and eruptive fluxes in particular generate radial stress fields^80^. Fluid flow is strongly controlled by fault systems that enable the transport of lithosphere- and mantle-derived volatiles to reach the surface where they discharge through volcanoes, spring systems or diffusely from the ground. Some volcanoes (e.g. Longonot^81^ and Aluto^41,82,83^; Figs. 1, 4) are dominated by structures formed during the current phase of rifting, including rift-parallel fault systems and volcanic structures such as caldera and crater ring faults. At other volcanoes (e.g. Corbetti; ref. ^84^ Figs. 1, 4), older, inactive faults which may be oblique or perpendicular to the current rift appear to control fluid pathways^85^.

## Past eruptions

Only ~25% of the Holocene EARS volcanoes have reported historical eruptions^80,86^ and for most EARS volcanoes, the date of the most recent eruption is still not known. Apart from a few exceptions e.g. refs. ^87,88^, historical accounts are poorly detailed, with very few accurate observations of pre-eruptive unrest, eruption duration, magnitude and style, or impact on local communities and environment.

### Eruption style

Volcanic rocks erupted along the EARS are invariably rich in alkali elements with a reduced oxygen fugacity^89,90^, resulting in a viscosity up to two orders of magnitude lower than their calc-alkaline counterparts for a given temperature^89,91^. This relatively low viscosity combined with high volatile contents^18^ has important implications for the outgassing potential of the magmas and thus eruption dynamics^19^. The effect of low viscosity of the magmas is most pronounced in the extreme mobility of the melilite-nephelinite lava flows of Nyiragongo, which have recorded speeds of up to 100 km/h^92^. The potential of fractures opening on the volcano’s southern lower flanks, as happened in 1977, 2002 and 2021, especially puts the urban area of Goma at risk^25,93^ and could potentially also lead to phreatic explosions from lava entering Lake Kivu or highly explosive phreatomagmatic activity if ascending magma were to interact with shallow groundwater and/or lake water^94^.

In the EARS, few historical eruptions have been documented in detail^25,36,95^, and even fewer have ground-based instrumental observations. The largest historical eruption in East Africa was probably the May 1861 CE eruption of Dubbi in Eritrea (Fig. 1e), and which may have resulted in the more than 100 fatalities in this very sparsely populated region^87^. Using a combination of remote sensing imagery, limited eyewitness accounts, and field observations at selected outcrops^87^, the eruptive volume of (basaltic) trachyandesite lava flows is estimated to be 1–3 km^3^. Reports of ash fall up to 300 km West of the volcano, and a depositional thickness of 4.5 m of trachyandesitic to trachytic pyroclastic deposits at 12 km from the summit and about 50 cm of ash fall at 50 km distance^87^, are consistent with at least a VEI or Magnitude 4 eruption.

A notable recent event was the 2011 eruption of Nabro, located off-axis on the Eritrean-Ethiopian border. This eruption generated an extremely SO_2_-rich, but seemingly ash-poor, plume that reached the lower stratosphere (ca. 15–17 km) and emitted a total of ca. 4.5 Tg of SO_2_, one of the largest emissions of volcanic SO_2_ since the 1991 eruption of Mount Pinatubo^96^. On the ground, the eruption generated two trachybasaltic-trachyandesitic lava flows, one extending to ca. 15 km, and a tephra blanket of unconstrained thickness^95,97^. Other notable historical eruptions have occurred in the Dabbahu Manda Hararo segment, associated with its 2005–2010 rifting episode^63^ and at Erta’ Ale, which has regular lava lake overflows and/or effusive eruptions from flank fissures. The most recent of these started in January 2017 and lasted at least 18 months^69^.

Due to the paucity of historical records, information on past eruptions from most volcanoes of the EARS, including many caldera systems, relies solely on the geological record. Widespread, thick pyroclastic units both on land and in lacustrine sequences document past explosive eruptions from the trachyte and phonolite volcanoes of Kenya and Tanzania^98–100^. Detailed mapping of the Rungwe Pumice deposit suggests it was formed by a Plinian eruption of Rungwe volcano with a column ca. 30 km above sea level and ash dispersal over >1000 km^2^ ^100^. Sustained explosive Plinian-style eruptions may also occur from peralkaline rhyolite magmas of the MER^15,101^.

Pumice cones have been identified at several EARS calderas^15,102–104^ and were in the past considered to be the rhyolite equivalent of strombolian scoria cones. However, a more detailed evaluation of fall deposits at Aluto^15,105^ and the pyroclasts embedded within them^102^ show that these pumice cones form during highly unsteady sub-Plinian eruptions, where the frequently collapsing plumes generate pyroclastic density currents. The proximal deposits gradually generate a cone-shape landform, which is often breached by a rhyolitic or obsidian lava flow towards the end of the eruption^102,103^. Individual phreatomagmatic vents, including tuff rings and maars, are known near many present-day large surface water bodies and/or important faults^16,106^, including in large fields of monogenetic cones and in the Manyara Rift, North Tanzania, and the Albertine Rift in Uganda^107^. Thus, the complex and multi-hazard nature of eruptions in the EARS needs to be considered when performing quantitative hazard assessments.

### Holocene eruption history

Establishing a frequency-magnitude relationship of Holocene eruptions requires constraints on absolute chronology, most commonly provided by radiocarbon dating of charcoal entrained within the deposits or in the underlying palaeosols^101,108^ and/or macrofossils or bulk sediment from lacustrine archives^15,105,109^. However, at many EARS volcanoes, little to no suitable material is preserved in the terrestrial sequences, partially because many studied deposits result from pyroclastic fall, which is less likely to trap and char vegetation, and/or because palaeosoils in several places (e.g. across the Ethiopian and Kenyan rifts) are organic-poor. Advancements in analytical precision of ^36^Ar–^39^Ar dating means that Holocene whole-rock or single phenocryst samples can now be dated, although the number of available dates for young deposits remains limited^98,110^.

**Fig. 5:**
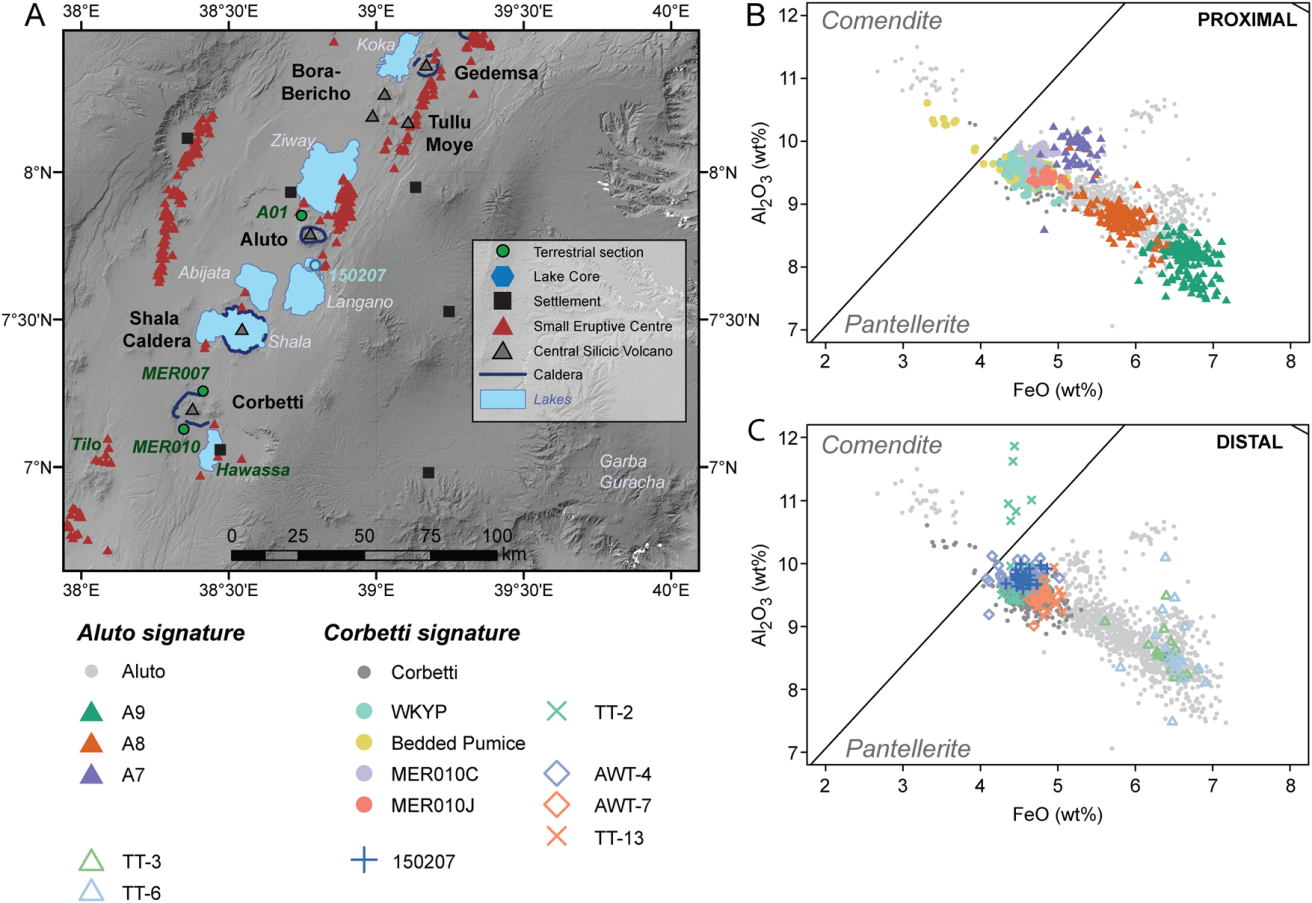
Geochemistry of Central Main Ethiopian Rift Volcanoes. **A** Map showing type sections of pyroclastic stratigraphy (green dots) and lake sediment cores (blue pentagons) near Aluto and Corbetti volcanoes in the Main Ethiopian Rift. All show multiple volcanic ash layers that can be attributed to Aluto or Corbetti and demonstrate the potential of developing a regional-scale multi-volcano tephrostratigraphic framework. The light green dot to the south of Aluto shows a locality of a pyroclastic deposit (150207) that has a Corbetti signature. Modified after^10^. **B** Chemical signatures of Aluto and Corbetti proximal deposits. **C** Chemical signatures of Aluto and Corbetti distal deposits The Corbetti signature plots as a cluster near 9–10 wt% Al_2_O_3_ and 4–5 wt% FeO, whereas the Aluto chemical compositions partially overlap this cluster, but span a much wider range and are thus easier to distinguish.

**Fig. 6:**
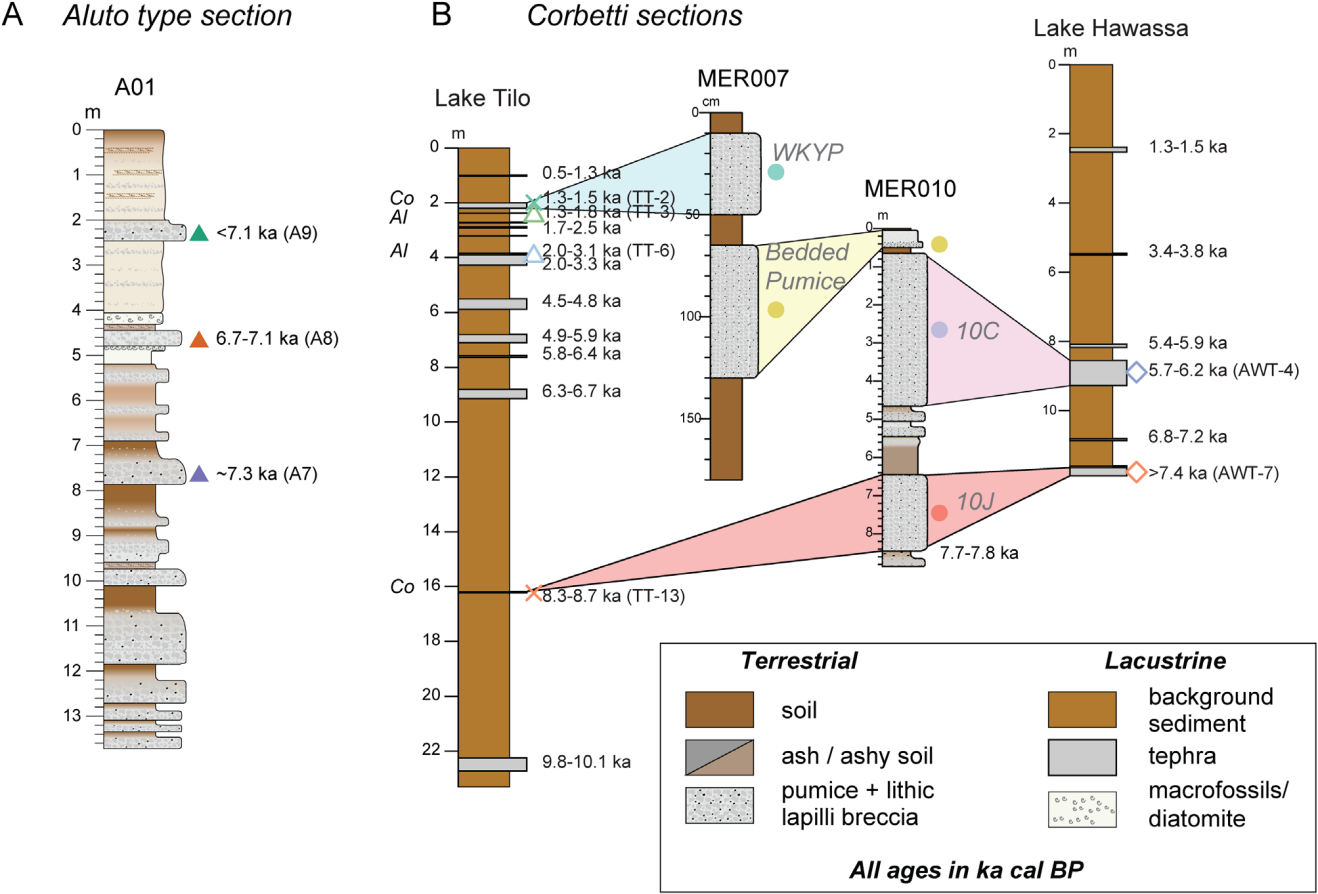
Eruption chronology of the Central Main Ethiopian Rift. **A** Schematic stratigraphic column of a terrestrial type section to the NW of Aluto showing a sequence of pyroclastic deposits, soils and lacustrine deposits, demonstrating a history of repeated explosive eruptions throughout the Holocene. **B** Schematic correlation between terrestrial and lacustrine stratigraphic columns near Corbetti. The Lake Tilo record shows predominantly ash layers of Corbetti signature, with the exception of two (TT-3 and TT-6) that have an Aluto signature. All other schematic logs on the figure have either only Aluto (type section A01) or Corbetti deposits (MER007, MER010 and Lake Hawassa). Co Corbetti, Al Aluto. Modified after ref. ^10^, with additional data from ref. ^108^. See Fig. 5 for details of locations and chemical compositions.

Lacustrine records often preserve ash deposits of moderate-intensity and/or distal eruptions, providing an important additional constraint on eruption recurrence intervals and the distal dispersal patterns of volcanic ash^105,109^. However, the complexity in eruptive style recorded by proximal terrestrial sequences is not detectable in the distal lacustrine records, making reconciliation challenging (Figs. 5, 6). Nonetheless, integrated studies of terrestrial-lacustrine archives have been conducted at several EARS volcanoes (e.g., Rungwe, Aluto, Corbetti). These show more frequent eruptions than were previously expected based on the historical record alone, with average recurrence intervals of the order of 10^2^–10^3^ years^15,17,109^.

At some volcanoes, e.g. the basaltic complexes in Afar, effusive eruptions dominate, and in such cases, mapping of lava flow units using high-resolution satellite imagery, sometimes combined with^36^Ar–^39^Ar chronology can provide constraints on the long-term eruptive rates of volcanic complexes^110–112^. For example, most of the Gudda-Bericha (also “Boset Volcanic Complex”, Fig. 1e) edifice is thought to have built above its MER rift floor in the last 30 ka, with activity alternating between the main centres of the complex within this timeframe^110^. The total estimated post-caldera volumes of Aluto and Corbetti relative to the age of their caldera-forming eruptions, yield long-term eruptive rates of order 0.08–0.09 km^3^/ka. At other volcanoes in the MER with lower post-caldera volumes, these rates may be much lower, though the age of their caldera-forming events is poorly constrained^16^. At Alu-Dalafilla (Fig. 1a), a complicated pattern of both fissure-dominated basaltic and central silicic eruptions emerges with a total erupted volume of the most recent edifices estimated to be between 0.5 and 2.4 km^3^, though absolute chronology is not constrained^112^. Despite these advances, reconstructing an integrated view of the effusive-explosive eruptive history at volcanic complexes remains challenging.

## Managing volcanic risks in sub-Saharan Africa

### Approaches to hazard assessment

Quantitative volcanic hazard assessment has rarely been applied in East Africa; the 2015 Global Assessment of Risk could only provide well-constrained hazard scores for 6% of volcanoes in the region^1^. Volcano-geological studies are critical for establishing frequency-magnitude relationships of past and therefore possible future eruptions (Fig. 6), but attempts at reconstructing a comprehensive eruption dataset for the EARS face several limitations including the limited absolute chronology and poor deposit preservation (see Section Holocene eruption history). Expert elicitation, which uses a series of guided questions to document a group of experts’ mental models of uncertain quantities, can be used to enhance quantitative estimates of frequency-magnitude relationships based on reconnaissance-style surveys alone^113,114^. This allows the inclusion of events for which no geological constraints exist, such as pre-historic eruptions of small magnitude that are not preserved and has been applied to several volcanoes in East Africa, including Aluto and Corbetti in Ethiopia^113,114^.

Probabilistic volcanic hazard assessment has previously been applied in East Africa to evaluate the conditional probability of inundation by lava flows^115^ or pyroclastic density currents^103^. This approach uses statistical tools to estimate vent opening location, and numerical models to simulate physical processes, where possible informed by field observations. At Aluto, the wide spatial distribution of vent locations, primarily constrained by the caldera ring fault, greatly influences (increases) the area that can potentially be impacted by pyroclastic density currents given a pumice cone eruption. The central caldera floor, home to an estimated 3000 people, a school, and a small geothermal facility, shows a 12% conditional probability of inundation by pyroclastic density currents given an eruption^103^. However, historical and geological observations show that EARS eruptions are more complex than conveyed in these simple models, and may involve multiple hazards.

A further step is to incorporate both volcanological data and dedicated hazard assessments into an integrated probabilistic event tree model that simulates conditional scenarios^116^. Even at Aluto, which could currently be considered the best-studied volcano in the EARS, i.e. with the most diverse dataset available, the epistemic uncertainty (largely driven by data availability) remains relatively large, with e.g. an estimated long-term probability of at least one silicic eruption at Aluto in the next 50 years between 2 and 35%^116^. However, the existing event tree model serves as a framework that can easily be adapted when new data become available (e.g. during an episode of volcano-seismic unrest or eruption).

In addition to understanding and quantifying physical hazards, their potential timescales and impact areas, a quantitative approach to understanding risk and risk perception is also essential. To this end, a precise view of population exposure and the development of population vulnerability indices can highlight strong spatial variations, as is the case in the city of Goma, exposed to volcanic hazards from Nyiragongo^117^. This information can then be combined with estimated impact areas of volcanic hazards (e.g. ref. ^115^) to better inform risk management and potentially identify priorities towards risk reduction strategies.

### Partnerships for developing resilience

Disaster risk reduction and climate change adaptation are key components of the UN Sustainable Development Goals, and governments across Africa are establishing frameworks to protect the lives of people and minimize property damage due to natural disasters. For example, Ethiopia has developed a multi-sector and multi-agency platform for disaster risk management; however, the emphasis is on food security because the long-recurrence intervals and lack of recent disasters caused by eruptions and earthquakes mean that geohazards are considered lower priority. Instead, investment in volcanology is focussed on the opportunities provided by geothermal power and geotourism.

Recent damaging events in the EARS serve as a useful reminder of the hazards associated with continental rifting, but do not reflect the potential impacts of a large magnitude earthquake or eruption in a densely populated region. For example, the 2011 eruption of Nabro occurred on the sparsely populated border between Ethiopia and Eritrea, but even so ~12,000 residents self-evacuated, at least 7 fatalities were recorded, two villages were completely destroyed and economic losses were estimated at $3 million^95^. Such events attract attention from the international scientific and aid communities, but this is not always accompanied by long-term investment in local capacity or monitoring facilities.

The East and Southern Africa Regional Seismological working group (ESARSWG) has been cooperating to compile a regional earthquake catalogue using data sourced from networks of permanent and temporary seismic stations in Eritrea, Ethiopia, Kenya, Uganda, Tanzania, Malawi, Zambia, Zimbabwe and Mozambique. However, eruption forecasting and alert systems require real-time analysis of small magnitude earthquakes. The Ethiopian national network provides real-time detection of moderate magnitude earthquakes (>M3.5) enabling rapid deployment of the local instrument pool and often attracting an international response^60,118^. While some real-time seismic stations exist elsewhere, most EARS countries rely on notifications from global systems with a higher detection threshold (>M4). Consequently, smaller events are often missed, and temporary seismic deployments have retrospectively catalogued thousands of small magnitude (0.5–3) events, low-frequency events and swarms characteristic of fluid movement^46,48^. While the development of automated algorithms and satellite systems may mitigate some challenges, unreliable power and telecommunications networks and numerous bureaucratic and logistical challenges mean improved resilience will require political and financial backing. Currently much of this relies on the support of international partners and limited-duration scientific projects^119,120^.

The Sendai Framework for Disaster Risk Reduction emphasizes the importance of understanding disaster risk and enhancing disaster preparedness. The UN Global Assessment of Risk included volcanoes for the first time in 2015^1^, and highlighted the challenges faced by many of the world’s least developed countries. In the EARS, recent damaging events combined with international research collaborations are driving a growing awareness of geohazards. In Ethiopia, the formation of a working group comprising government institutions, civil protection and infrastructure groups, led the National Disaster Risk Management Commission in 2019 to publicly announce ‘*a paradigm shift from managing geohazard crises to managing risks*’. In Tanzania, the 2007–2008 eruption of Oldoinyo Lengai triggered donor-supported public awareness projects at the universities and public institutions. Sensitization, thereafter, has been done regularly to reduce risk of death and property loss in case of earthquake and volcanic hazards. However, with a few notable exceptions, research in the EARS has been led by international partners aiming to further scientific understanding through reconstructing past events, rather than developing hazard assessments, monitoring networks or scenario planning. While co-designed partnerships with overseas researchers can provide a strong evidence base and strengthen capacity, African institutes bear the responsibility for managing the crises and risks. Thus there is an urgent need to further build human and institutional capacity through multi-disciplinary involvement and funding of academics in African universities, research institutions and geological surveys^121^.

## Conclusions and future directions

The volcanoes of the East African Rift System remain understudied in comparison to many subduction zone volcanoes. Multi-disciplinary studies over the past two decades have unearthed a rich history of volcanic activity and unrest in the EARS, providing new insights into characteristics of the volcanic plumbing systems and the foundation for hazard and risk assessments. Despite this, datasets remain comparatively sparse and many fundamental scientific and societal questions remain unanswered. For example, what drives volcanic eruptions over human and geological timescales, what controls the location of active magmatic systems and their unrest and what are the potential threats from future volcanic activity? The raised awareness of volcanic hazards is driving a shift from crisis response to reducing disaster risks, but a lack of institutional and human capacity means managing and mitigating geohazards in sub-Saharan Africa remains challenging. In this section, we briefly summarize some of the remaining scientific challenges associated with volcanism in continental rifts, and the EARS in particular.

Over the past decade, we have been able to image transcrustal magma plumbing systems at various stages of rift development. Vertically, these show the focussing from broad zones of partial melt in the upper mantle to shallow crustal reservoirs which feed basaltic dyke intrusions, or long-lived silicic reservoirs. Transects across the rift show that the deeper parts of this system are often laterally offset from the rift axis (Fig. 2) raising further questions regarding the nature of volcanism along the rift margins and flanks and the connectivity between regions of partial melt both along- and across-rift.

Another major development over the past decade has been the development of eruption chronologies based on geological records from lake sediments, terrestrial sections and lava flow mapping. However, in most cases these remain disconnected, and a full understanding of the magnitude-frequency relationships will require reconciliation of on-shore and offshore records, and of records of explosive and effusive activities. Some eruption styles, such as monogenetic or phreatic eruptions, are poorly recorded in the geological record, and thus challenging to integrate within this framework, despite their prevalence in analogue environments.

Satellite monitoring and short-term instrument deployments have demonstrated that many of the caldera systems in the EARS are currently experiencing unrest, and awareness of volcanic hazards is rising. However, there remain many scientific and societal barriers to developing a forecasting or alert level system capable of responding to changes in behaviour. The available geophysical records provide a snapshot of activity and the vast majority of volcanic systems in the EARS still lack even the most basic monitoring instrumentation. Only long-term, multi-disciplinary baseline monitoring integrating ground-based and satellite observations will be capable of distinguishing between background, elevated and escalating states of unrest. In the past few years, enough data have become available to attempt the very first probabilistic hazard assessments and these provide a framework for future studies. However, each volcano has distinct behaviour controlled by the complex interplay between stress, rheology and fluid flow and driven by a range of poorly understood tectonic, magmatic and climate processes. Characterizing each system represents an enormous data gathering challenge followed by interpretations that eventually lead to physical-chemical volcano models, which can only be met by developing infrastructure, expertise and education within African Institutions.
